# Cohort profile: Cardiovascular Metabolic Etiological Research Center COVID-19 Mental Health Survey (CC-MHS)

**DOI:** 10.4178/epih.e2025033

**Published:** 2025-06-30

**Authors:** Sun Jae Jung, Dongkyu Lee, Ji Su Yang, Sunghyuk Kang, Hyejin Kim, Jeong Hyun Ahn, Yunseong Heo, Jieun Noh, Changhyun Kim, Hyeon Chang Kim

**Affiliations:** 1 Department of Preventive Medicine, Yonsei University College of Medicine, Seoul, Korea; 2 Department of Public Health, Yonsei University College of Medicine, Seoul, Korea; 3 Jeonbuk National University Medical School, Jeonju, Korea

**Keywords:** COVID-19, Mental health, Cohort, Post-traumatic stress disorder, Depression, Anxiety

## Abstract

The Cardiovascular Metabolic Etiological Research Center COVID-19 Mental Health Survey (CC-MHS) is a comprehensive longitudinal cohort study investigating the mental health impact of the coronavirus disease 2019 pandemic by utilizing pre-existing baseline data from the Cardiovascular Metabolic Etiological Research Center cohort (2013-2018). This study assesses physical health, lifestyle changes, and mental health using validated tools, including the Patient Health Questionnaire-9, Generalized Anxiety Disorder-7, and the PTSD Checklist for DSM-5, and evaluates a population of urban and suburban Korean participants across multiple dimensions. Through online surveys, the research identified gender-specific social support mechanisms, showing that men benefit from larger social networks, whereas women derive protective effects from stronger emotional connections. Key findings underscore complex interactions among demographic factors, psychological variables, and public health responses, especially in the context of vaccination attitudes and trust in pandemic management. The CC-MHS delivers critical insights into mental health trajectories during global health crises, offering valuable evidence for developing adaptive public health strategies and for understanding the intricate relationships between individual psychological resilience and broader societal health challenges.

## INTRODUCTION

The coronavirus disease 2019 (COVID-19) pandemic has precipitated a global public health emergency with mental health implications that extend well beyond immediate concerns of viral transmission. Epidemiological studies indicate a substantial mental health burden, as demonstrated by a systematic review and meta-analysis [1] reporting pooled global prevalence estimates for psychological impacts: anxiety (6.3-50.9%), depression (14.6-48.3%), and post-traumatic stress disorder (PTSD; 7.0-53.8%). Cross-sectional research from China [2] further substantiated these findings, revealing that 53.8% (95% confidence interval [CI], 52.3 to 55.3) of respondents rated the psychological impact of the outbreak as moderate or severe. Consistent with this increased prevalence, the pandemic was estimated to add 10.70 million and 9.05 million disability-adjusted life-years to the global burden due to depressive and anxiety disorders, respectively, representing over a quarter of the pre-pandemic burden [3].

Retrospective longitudinal analyses involving 236,379 COVID-19 survivors [4] have provided compelling evidence for the pandemic’s long-term mental health consequences. These studies demonstrated significantly increased hazard ratios (HRs) for psychiatric conditions relative to matched controls: anxiety disorders (HR, 1.35; 95% CI, 1.30 to 1.39), depressive disorders (HR, 1.39; 95% CI, 1.34 to 1.44), and stress and adjustment disorders (HR, 1.38; 95% CI, 1.34 to 1.43). Such findings highlight the profound and potentially enduring psychological sequelae associated with COVID-19.

Despite these findings, major research limitations persist, particularly the lack of comprehensive baseline data predating the pandemic. This methodological gap restricts researchers’ ability to accurately assess changes in mental health trajectories, monitor long-term outcomes, and identify vulnerable subgroups. While several international cohorts—including those in the Netherlands [5], Estonia [6], Norway [7], and Iceland [8]— have utilized pre-existing data to study pandemic-related mental health impacts, such research remains notably sparse in Asian contexts and is characterized by predominantly short-term follow-up periods [9].

The present study aims to address these critical gaps by longitudinally evaluating the mental health impact of COVID-19 using an established community cohort. By examining a comprehensive range of mental health aspects and related variables both during and after the pandemic, this research aims to elucidate how baseline physical, mental, and social characteristics collectively shape individual resilience and vulnerability. A particular strength of this study is its use of in-depth, repeated mental health assessments before and after the COVID-19 pandemic, enabling a detailed examination of residual mental health issues in the post-pandemic period.

The significance of this research extends beyond academic interest. Understanding these complex interactions is vital for developing targeted, evidence-based interventions, informing public health policy, and enhancing health system preparedness for future global health crises. By clarifying the intricate relationships between individual characteristics and pandemic-induced psychological stress, this study provides a critical framework for building resilience at both the individual and community levels in the face of unprecedented global challenges.

## STUDY PARTICIPANTS

The Cardiovascular Metabolic Etiological Research Center (CMERC) cohort, established from 2013 to 2018, enrolled 8,097 participants from urban and suburban areas around Seoul, Korea. The cohort included residents from Seoul, Incheon, and Gyeonggi Province, and comprised both community-based low-risk and hospital-based high-risk populations. Recruitment was conducted through newspaper advertisements, public posters, and referrals from existing participants. Eligibility criteria for the community-based cohort included individuals aged 30-64 years who were community-dwelling, non-pregnant, and free of major cardiovascular diseases such as myocardial infarction or heart failure. Additional requirements included a minimum residency of 8 months at their current address and no intention to relocate within 2 years. These participants formed the baseline population for the Cardiovascular Metabolic Etiological Research Center COVID-19 Mental Health Survey (CC-MHS). Of these, 3,913 individuals provided consent to be contacted and participated in the CC-MHS online mental health follow-up surveys beginning in 2020.

### Baseline Cardiovascular Metabolic Etiological Research Center data of 4,060 participants

The baseline data for the 4,060 participants included a broad array of variables: demographic characteristics; physical health assessments (including anthropometry, blood pressure, electrocardiogram, carotid ultrasound, fasting blood tests, and urinalysis); health behaviors (physical activity, diet, smoking, alcohol consumption); medical history; and psychosocial measures. Psychosocial assessments included depression (using the Beck Depression Inventory-II), stressful life events (via the Life Experiences Survey), cognitive function (using the Korean version of the Mini-Mental State Examination–Dementia Screening for individuals aged 50 years and older), and social network properties (such as network size, density, emotional closeness, and contact frequency).

### Structured interviews and online surveys

Structured interviews were conducted as part of the CMERC cohort’s initial baseline assessments from 2013 to 2018. These interviews collected detailed data on medical history, lifestyle factors, and psychosocial variables. The CC-MHS surveys, launched in 2020, relied exclusively on self-administered online questionnaires and did not include new structured interviews.

### Timeline and baseline definition

Although CC-MHS mental health data collection began in March 2020 during the early phase of the COVID-19 pandemic, the study leveraged extensive pre-pandemic baseline data obtained from the CMERC cohort (2013–2018). The CC-MHS thus enables a longitudinal evaluation of mental health trajectories across 3 distinct periods: pre-pandemic (as defined by the CMERC baseline), pandemic onset and progression (as captured by CC-MHS follow-up surveys), and the post-pandemic period (via ongoing CC-MHS follow-up).

#### CC-MHS

Following the COVID-19 outbreak in January 2020, the CC-MHS was launched to assess both the short-term and long-term mental health impacts of the pandemic. The mental health sub-cohort consisted of 4,060 urban residents in the Seoul metropolitan area, with participants receiving the baseline survey in March 2020 (Wave 1). Invitations were sent to 3,913 individuals who had previously consented to the study, along with an explanatory document detailing the substudy’s purpose and methodology, delivered by postal mail. A follow-up text message containing the survey link was sent to each participant’s personal mobile phone, with a second reminder message sent to those who did not respond initially. A US$10 gift voucher was provided to participants upon survey completion.

**Figure 1.**
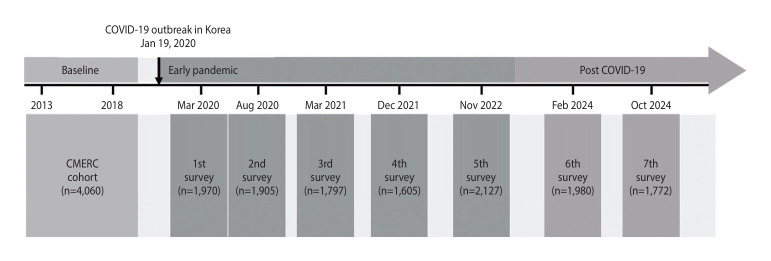
History of the CMERC COVID-19 Mental Health Survey (CC-MHS) establishment and follow-up. CMERC, Cardiovascular Metabolic Etiological Research Center; COVID-19, coronavirus disease 2019.

In addition to the baseline survey, 7 waves of online mental health surveys were conducted at intervals of approximately 6 months to 1 year: Wave 2 in August 2020, Wave 3 in March 2021, Wave 4 in December 2021, Wave 5 in November 2022, Wave 6 in February 2024, and Wave 7 in October 2024. The study has maintained an active follow-up design, ensuring sustained participant engagement and robust data collection. The overall response rate was 71.80% for participation in at least 1 wave, with wave-specific response rates of 48.52% (Wave 1), 46.92% (Wave 2), 44.11% (Wave 3), 39.53% (Wave 4), 52.39% (Wave 5), 48.77% (Wave 6), and 43.67% (Wave 7). This comprehensive longitudinal approach allows for a detailed analysis of the mental health implications of COVID-19 over time, with data collection still ongoing (Figure 1).

Out of the 8,097 individuals in the CMERC cohort, 4,060 were eligible for the CC-MHS, and 3,913 individuals consented to participate in the online mental health surveys, corresponding to approximately 48% of the original CMERC cohort. The participation rate across follow-up waves varied, with 71.80% of eligible participants completing at least 1 wave. As noted, some degree of selection bias may exist, as participants tended to be relatively healthier and more interested in mental health research.

### Ethics statement

Institutional review board approvals were obtained from Severance Hospital, Yonsei University Health System, and written informed consent was secured from all participants prior to baseline assessments [10].

This study adheres to the ethical principles outlined in the Declaration of Helsinki. Informed consent was obtained from all participants. Both the baseline evaluation and subsequent online follow-up surveys received approval from the relevant institutional review board (CMERC: 4-2013-0661; CC-MHS: Y-2020-0066, Yonsei University Health System).

## MEASUREMENTS

A wide range of data, including genetic and telomere information, was collected from the cohort. At baseline, participants underwent structured interviews covering demographic characteristics, medical history, health behaviors, psychological health, and social network attributes. Physical activity was assessed using the Korean version of the International Physical Activity Questionnaire, and chronic disease history was self-reported. Social network properties were evaluated by asking participants to identify their network members and provide information on network size, density, emotional closeness, and contact frequency.

Anthropometric and clinical assessments included measurements of body composition, blood pressure, electrocardiogram, carotid artery ultrasonography, fasting blood tests, and urinalysis. Mental health evaluations comprised assessments of depressive symptoms (Beck Depression Inventory-II), stressful life events (Life Experiences Survey), and cognitive function (Mini-Mental State Examination-Dementia Screening, for those aged 50 and older). The study protocol has been described in detail in previous publications [11].

The CC-MHS provides a comprehensive dataset that captures longitudinal health outcomes, behavioral trends, and the multifaceted effects of the COVID-19 pandemic, as measured over 7 waves from March 2020 to October 2024. Table 1 summarizes the survey items collected at each wave. The cohort size began with 1,970 individuals in the first survey, with subsequent participation reflecting dynamic changes over time, including a decrease to 1,605 participants in the fourth survey and an increase to 2,127 in the fifth. These fluctuations illustrate evolving participation patterns and the study’s adaptive approach to capturing relevant data throughout different phases of the pandemic.

Physical health assessments relied on self-reported measures of height and weight, along with participants’ disease history and treatment status. Although direct clinical measurements were not conducted, these self-reported data provided valuable insights into chronic health conditions and overall trends in physical health over time. Participants also reported changes in key lifestyle factors, including diet, physical activity, sleep patterns, and alcohol or cigarette use. These variables allowed the study to evaluate the impact of the pandemic on health-related behaviors and their potential role in shaping health outcomes.

Mental health outcomes were rigorously assessed using validated psychometric tools, including the Patient Health Questionnaire-9 (PHQ-9) for depression [12], Generalized Anxiety Disorder-7 (GAD-7) for anxiety [13], the PTSD Checklist for DSM-5 (PCL-5) [14], the UCLA Loneliness Scale (ULS-6) [15], and the Connor-Davidson Resilience Scale (CD-RISC-10) [16]. Each instrument has undergone validation: the PHQ-9, GAD-7, and PCL-5 specifically in Korean populations, the ULS-6 across multiple populations, and the CD-RISC-10 in American populations. Consistent assessments of suicide risk were included, and non-suicidal self-injury was measured from the fourth survey onward. Collectively, these instruments provided a robust framework for monitoring mental health trajectories and exploring their associations with pandemic-induced stressors, lifestyle changes, and resilience factors. Table 2 presents the basic characteristics of the follow-up surveys.

COVID-19-related status items formed a central part of the survey, capturing various facets of the pandemic’s impact on individuals. These included changes in healthcare utilization, such as delayed or reduced hospital visits. The survey also assessed participants’ perceptions of the pandemic, including their understanding of disease severity, risk perception, and coping strategies. Particular emphasis was placed on attitudes toward COVID-19 vaccination, evaluating trust in vaccine safety and efficacy as well as willingness to be vaccinated. These measures yielded important insights into the drivers of vaccine acceptance and hesitancy, offering crucial data for guiding public health interventions.

The survey further examined participants’ trust in national and community-level pandemic responses and explored experiences of stigma related to COVID-19. Items addressed trust in government policies, public health measures, and community support systems, as well as stigma experienced due to exposure, diagnosis, or vaccination status. In addition, changes in socioeconomic status (SES) were assessed, with participants reporting alterations in employment, income, and financial stability during the pandemic. These variables captured the broader societal effects of COVID-19, illuminating how economic disruptions influenced health and well-being.

Overall, the CC-MHS constitutes a rich and multidimensional dataset for investigating the complex interplay among physical and mental health, lifestyle factors, socioeconomic changes, and social dynamics in the context of the COVID-19 pandemic. By including variables such as trust, stigma, lifestyle changes, and SES fluctuations, the study provides critical insights into the determinants of individual and community resilience during an unprecedented period of global disruption.

## KEY FINDINGS

The CC-MHS initiated recruitment in 2020, with several publications emerging from this ongoing longitudinal study. The key findings are described below.

Initial research published in 2021 evaluated the impact of COVID-19 on mental health, with a particular focus on pre-existing depression status. Analysis of CMERC cohort data revealed significant mental health effects moderated by gender and baseline mental health conditions. The study identified social support and access to essential supplies as important protective factors, especially for individuals with prior depression who demonstrated increased vulnerability. These results underscore the necessity for tailored pandemic interventions to address mental health disparities [17].

Research into information-seeking behaviors during the COVID-19 pandemic revealed their dual impact. While such behaviors promoted preventive actions—such as mask-wearing and social distancing—they also heightened anxiety and fear, leading to negative outcomes like hoarding and the adoption of unverified remedies. These findings highlight the critical role of information both in supporting public health and in potentially exacerbating psychological distress [18].

A 2022 study applied joint latent space item response models to explore stressful life events (SLEs) and their association with depressive symptoms. The analysis identified distinct patterns linking specific SLEs to depressive symptoms by gender and age group, offering new insights into life event–mental health interactions and suggesting the value of demographically targeted interventions [19].

Another study in 2022 investigated trust and intention regarding COVID-19 vaccination using community-based longitudinal data. The results demonstrated that socio-demographic factors, mental health status, and psychological resilience were associated with vaccine trust and intention. Of note, the study found concerningly low trust levels in certain demographic groups, highlighting the importance of targeted public health strategies to increase vaccine acceptance [20].

Research published in October 2023 examined the association between social networks and post-traumatic stress disorder (PTSD) symptoms during the pandemic in a Korean community-based cohort. The findings showed gender-specific protective effects: larger social networks were associated with reduced PTSD symptoms in men, while emotional closeness had a stronger protective effect in women. These results emphasize the crucial role of social networks in crisis-related psychological resilience and support the need for gender-specific interventions [21].

Collectively, these studies demonstrate the multidimensional effects of the COVID-19 pandemic on mental health, behavior, and public health responses. They underscore the importance of psychological resilience, equitable access to resources, and effective communication strategies in crisis management. The findings highlight the significance of social support, tailored interventions, and trust-building for mitigating the impacts of the pandemic.

## STRENGTHS AND WEAKNESSES

The CC-MHS is a longitudinal cohort study that has continuously tracked the mental health of community-dwelling individuals from before the onset of the COVID-19 pandemic through the post-pandemic period. This extended follow-up provides a unique opportunity to assess long-term mental health trajectories across different pandemic phases. Unlike many previous studies, this cohort allows for a comprehensive evaluation of the pandemic’s impact, as few have systematically followed individuals both before and after its emergence. The cohort leverages robust pre-pandemic baseline data from the CMERC cohort (2013-2018) and conducts repeated mental health assessments from the early COVID-19 period (March 2020) through the post-pandemic era. This design uniquely enables longitudinal evaluations of mental health trajectories, accounting for the influences of individual, social, and pandemic-related factors. The availability of comprehensive pre-pandemic baseline data, including biomarkers and detailed health assessments, increases the ability to identify both residual and long-term mental health impacts of the pandemic. In addition to COVID-19-specific factors, the cohort comprehensively examines both physical and mental health variables, providing a multidimensional perspective on health outcomes. The study offers valuable data for observing changes in population mental health during a social disaster, owing to its foundation in pre-existing baseline data with key biomarkers and physical measurements. This aspect is particularly important, as many other COVID-19 mental health cohort studies lacked such comprehensive baseline information. Furthermore, the cohort enables long-term assessment of the pandemic’s impact on both individual and community health, offering critical insights into how social, economic, and health-related factors interact over time. It also serves as a valuable resource for investigating post-pandemic residual mental health issues, which have not been extensively addressed in prior research. The findings published from this cohort are of considerable public health significance and have been compared with results from international studies. Additionally, the CC-MHS serves as a critical resource for understanding interactions between socioeconomic factors—such as social networks and social support—and mental and physical health outcomes over time.

Nonetheless, the cohort has certain limitations. Although it includes participants from the Seoul and Gyeonggi regions, it is not population-representative. Individuals originally enrolled in the CMERC study were generally healthier and had relatively higher income levels. Moreover, those who voluntarily participated in the CC-MHS may have a heightened interest in mental health and tend to be healthier, potentially introducing a healthy volunteer bias. Since the study is online-based, there may have been selection bias due to differences in digital accessibility. However, with a mobile phone penetration rate exceeding 99% in Korea, the likelihood of significant bias from this factor is relatively low. The low participation rate relative to the initially eligible population raises concerns about selection bias, which may have reduced the study’s statistical power. Additionally, as an internet-based survey, the study could not assess certain measures—such as anthropometric data—that require direct, in-person evaluation. Despite these limitations, the cohort maintained a relatively consistent population size throughout the follow-up period. Some attrition was observed for certain variables, which could also contribute to selection bias. Another limitation is that the cohort size is not large enough to comprehensively study suicide-related behaviors. To address this, the study has joined the international COVID-19 Global Mental Health Consortium to enhance analytical power. These limitations underscore the need for larger-scale follow-up studies focused on similar topics to yield more robust and generalizable insights. The cohort’s limited representativeness, potential healthy volunteer bias, and lower participation rate relative to the original CMERC sample warrant cautious interpretation. These limitations highlight the importance of complementary studies and collaborations, such as participation in the COVID-19 Global Mental Health Consortium, to enhance generalizability and analytical robustness.

## DATA ACCESSIBILITY

The cohort data are not freely available, but the CC-MHS team welcomes collaborations with other researchers. For further information, contact Dr. Jung (the corresponding author).

## Figures and Tables

**Table 1.** Survey items of the CMERC COVID-19 Mental Health Survey (CC-MHS)

Surveys	1st	2nd	3rd	4th	5th	6th	7th
Survey date	Mar 2020	Aug 2020	Mar 2021	Dec 2021	Nov 2022	Feb 2024	Oct 2024
No. of participants	1,970	1,905	1,791	1,605	2,127	1,980	1,772
Questionnaire items							
Physical health							
Disease history	X	X	X	X	X		
Disease treatment history					X		
Anthropometrics						X	
Mental health							
PHQ-9	X	X	X	X	X	X	X
GAD-7	X	X	X	X	X	X	X
PCL-5	X	X	X	X	X	X	X
ULS-6	X	X	X	X	X	X	X
CD-RISC-10	X	X	X	X	X	X	X
Suicidality	X	X	X	X	X	X	X
Sleep duration/disturbance	X						
PSQI		X	X	X	X	X	X
Psychiatric history		X	X	X	X	X	X
Mental health treatment history			X	X	X	X	
Mental health care utilization						X	
Experiences with mental health treatment						X	
COVID-19-related medical experience							
Change in frequency of hospital visits		X	X				
Barriers in treatment			X				
Other COVID-19-related statuses							
COVID-19 infection history	X	X	X	X	X	X	
Concerns during isolation and quarantine	X						
Subjective perception of COVID-19	X	X	X	X	X	X	
Fear of infection due to COVID-19	X	X	X	X	X		
Preventive actions against COVID-19	X	X	X				
COVID-19-related information acquisition (method, time spent, satisfaction)	X	X	X				
Projections of COVID-19 spread and impact			X	X	X		
Challenges associated with lifestyle changes			X	X		X	
Long COVID symptoms					X		
Social support							
Access to material, psychological, and daily living support	X	X	X				
Caregiving responsibilities		X	X				X
Transformations in age-related and familial social norms							X
Governmental responses to demographic shifts							X
Intergenerational dynamics and obligations							X
Attitudes toward marriage and family formation							X
Evaluating the impact of population policies							X
Stigma							
Self-stigmatization following infection	X			X	X	X	
Social stigma and discrimination against infected individuals		X	X	X	X	X	
Public Trust in National and Local Institutions							
Public satisfaction with governmental responses	X	X	X				
Attitudes toward the ‘Living with COVID-19’ strategy				X	X		
COVID-19 vaccine							
Perceived efficacy and trust in COVID-19 vaccines			X	X		X	
Willingness to receive the COVID-19 vaccine			X	X			
Motivations and barriers to vaccination				X			
No. of vaccinations						X	
Perceived risks and safety concerns of vaccination						X	

CMERC, Cardiovascular Metabolic Etiological Research Center; COVID-19, coronavirus disease 2019; PHQ-9, Patient Health Questionnaire-9; GAD-7, Generalized Anxiety Disorder-7; PCL-5, PTSD Checklist for DSM-5; ULS-6, UCLA Loneliness Scale; CD-RISC-10, Connor-Davidson Resilience Scale; PSQI, Pittsburgh Sleep Quality Index.

**Table 2.** Baseline characteristics of participants in the CMERC COVID-19 Mental Health Survey (CC-MHS)

Surveys	1st	2nd	3rd	4th	5th	6th	7th
Survey date	Mar 2020	Aug 2020	Mar 2021	Dec 2021	Nov 2022	Feb 2024	Oct 2024
No. of participants (n)	1,970	1,905	1,791	1,605	2,127	1,980	1,772
Age, mean±SD (yr)	54.99±9.22	55.47±9.26	56.00±9.35	56.71±9.36	57.94±9.29	59.75±9.12	60.11±9.06
Gender							
Men	693 (35.2)	655 (34.4)	626 (34.9)	572 (35.6)	760 (35.7)	665 (33.6)	608 (34.3)
Women	1,277 (64.8)	1,250 (65.6)	1,165 (65.0)	1,033 (64.4)	1,367 (64.3)	1,315 (66.4)	1,165 (65.7)
COVID-19 infection history^1^							
Confirmed case	2 (0.1)	5 (0.3)	8 (0.4)	N/A	N/A	N/A	N/A
Suspected case	6 (0.3)	3 (0.2)	33 (1.8)	N/A	N/A	N/A	N/A
Self-quarantine	12 (0.6)	20 (1.0)	0 (0)	N/A	N/A	N/A	N/A
Active surveillance	21 (1.1)	7 (0.4)	8 (0.4)	N/A	N/A	N/A	N/A
Not applicable	1,872 (97.9)	1,869 (98.2)	1,737 (97.3)	N/A	N/A	N/A	N/A
Never infected	N/A	N/A	N/A	1,572 (98.4)	787 (37.3)	448 (23.0)	N/A
Ever infected	N/A	N/A	N/A	25 (1.6)	1,321 (62.7)	1,496 (76.9)	N/A
Mental health							
Depression (PHQ-9)	2.72±3.75	4.04±4.44	5.05±5.16	4.59±4.99	3.09±4.26	3.51±3.87	3.48±3.94
Anxiety (GAD-7)	4.25±4.45	2.48±3.43	2.93±3.98	2.77±3.84	1.91±3.16	2.09±3.01	2.24±3.11
PTSD (PCL-5)	10.29±10.25	7.95±9.31	9.92±12.00	10.47±12.68	7.83±10.32	8.32±9.80	8.83±10.24
Loneliness (ULS-6)	10.12±3.07	10.54±3.20	10.58±3.44	10.39±3.34	9.70±3.09	9.69±3.08	9.90±3.26
Resilience (CD-RISC-10)	26.93±8.90	25.20±8.81	25.31±8.50	25.77±8.08	24.75±10.09	25.74±9.21	25.75±9.50
Suicidality	18 (0.9)	30 (1.6)	80 (4.5)	47 (2.9)	60 (2.8)	48 (2.4)	52 (2.9)

Values are presented as number (%) or mean±standard deviation. CMERC, Cardiovascular Metabolic Etiological Research Center; COVID-19, coronavirus disease 2019; N/A, not available; PHQ-9, Patient Health Questionnaire-9; GAD-7, Generalized Anxiety Disorder-7; PCL-5, PTSD Checklist for DSM-5; ULS-6, UCLA Loneliness Scale; CD-RISC-10, Connor-Davidson Resilience Scale.

1 1st-3rd surveys: Current COVID-19 diagnosis and isolation status; 4th-6th surveys: COVID-19 diagnosis throughout the pandemic period.
